# Oxidation processes related to seed storage and seedling growth of *Malus sylvestris, Prunus avium* and *Prunus padus*

**DOI:** 10.1371/journal.pone.0234510

**Published:** 2020-06-18

**Authors:** Mikołaj Krzysztof Wawrzyniak, Ewa Marzena Kalemba, Ewelina Ratajczak, Paweł Chmielarz

**Affiliations:** Institute of Dendrology, Polish Academy of Sciences, Kórnik, Poland; Brigham Young University, UNITED STATES

## Abstract

Seeds stored in controlled conditions in gene banks, faster or slower lose their viability. The effects of seed moisture content levels (ca. 5, 8, 11%) combined with storage temperatures (-3°, -18°, -196°C) were investigated in terms of the description of seeds defined as orthodox under oxidative stress after seed storage, during germination, and initial seedling growth. Hydrogen peroxide (H_2_O_2_), thiobarbituric acid reactive substances (TBARS) and ascorbate (Asc) were analyzed in relation to seed germinability and seedlings emergence in three species: *Malus sylvestris* L., *Prunus avium* L. and *Prunus padus* L. The effect of seed storage conditions on H_2_O_2_ levels appeared in germinated seeds after the third year of storage in each species. The H_2_O_2_ levels were negatively correlated with the germination and seedling emergence of *P*. *avium* seeds after three years of storage under all examined combinations. The emergence of *P*. *padus* seedlings was not linked to any of the stress markers tested. The *P*. *padus* seed biochemical traits were least altered by storage conditions, and the seeds produced tolerant seedlings of relatively high levels of H_2_O_2_ and TBARS. To cope with different H_2_O_2_ levels, TBARS levels, and Asc levels in seeds of three species varying storage conditions different molecular responses, i.e. repairing mechanisms, were applied during stratification to compensate for the storage conditions and, as a result, seeds remained viable and seedlings were successfully established.

## Introduction

*Ex situ* seed banking is an efficient and cost-effective method for preserving the genetic diversity of plants in the fast-changing world [1]. For seeds that tolerate desiccation and storage at sub-zero temperatures (i.e. orthodox seeds) the longevity of stored seeds depends on species biology and storage conditions such as moisture content and temperature [2]. Although some seeds can remain viable even over a thousand years in natural conditions [3,4], other species deteriorate relatively quickly even under optimal and controlled conditions [1,5]. Knowledge about unique species-related seed characteristics is valuable for long-term and effective storage in seed banks. One of the main factors controlling the deterioration of seeds during storage is the accumulation of reactive oxygen species (ROS), e.g., superoxide anion radicals (O_2_^•-^), hydrogen peroxide (H_2_O_2_) or hydroxyl radicals (•OH), as their production depends on the metabolic and physiological state of seeds during storage [6,7].

Wild fruit trees and shrubs make great contributions in European forest ecosystems. They serve as a source of nutrition, pollination or genetic resources for commercial breeding programs, enriching biodiversity and therefore ecosystem resilience [8]. However, due to the small economic importance of these species, they are often neglected in conservation programs. *Malus sylvestris* is considered to be endangered in only a few European countries (i.e. Belgium and the Czech Republic), despite its rare occurrence and population losses across the whole of Europe [9]. In addition to threats such as habitat fragmentation and low natural regeneration, some wild species can also suffer from genetic swamping by hybridizing with commercially cultivated varieties [10–12]. To preserve the genetic diversity of these species, effective seed storage protocols are required. Seeds of *M*. *sylvestris*, *P*. *avium*, and *P*. *padus* are considered orthodox, withstanding severe desiccation (below 5% moisture content) [13]. However, their storability under ultralow temperatures and moisture contents can differ from expectations. Wawrzyniak et al. [14] conducted studies on the effects of seed storage under different moisture content levels and temperatures of the above mentioned species. They established that seeds of *M*. *sylvestris*, despite being categorized as orthodox, lose viability when stored below 5% seed moisture content after first year of storage. In the case of *P*. *avium*, seedling emergence was strongly affected by storage in liquid nitrogen (-196°C; LN) despite a high germination percentage [15,16]. On the other hand, neither moisture content nor storage temperature affected the seeds of *P*. *padus* after three years of storage. It was uncertain what caused the loss in viability during storage under a low moisture content and temperature in the three orthodox seeded species. Low water content and low temperature promote glassy state formation [17,18], however several mechanisms of seed aging exist in terms of moisture content and temperature [19] and solid-state properties vary among tissues in seeds [18]. The removal of structural water strongly bounded to macromolecules, larger pores in the glassy matrix, and the exposure of reaction centers to ROS molecules contribute to higher oxidative stress [17].

H_2_O_2_ can act both as a signaling molecule and a toxin, depending on its concentration in the cell. The production of H_2_O_2_ occurs mainly in mitochondria, is greatly intensified after seed imbibition and further increases during the progression of the germination process [20]. As a signaling molecule, H_2_O_2_ directly affects dormancy release, endosperm weakening, and pathogen protection and interacts with other molecules and phytohormones [21]. However, H_2_O_2_ is also synthetized and accumulated in dry seeds during storage as a result of nonenzymatic reactions such as lipid peroxidation or Amadori and Maillard reactions [22,23]. H_2_O_2_ easily migrates through intercellular membranes, reaching target molecules even over a relatively long distance [24]. High concentrations of H_2_O_2_ have a deleterious effect on lipids (notably membranes and reserve lipids), proteins, DNA and RNA in the cell. DNA oxidation damage occurs during seed storage and germination [21,25,26]. Protein oxidation leads to the modification of their the enzymatic and binding functions of protein, causing their malfunction [27]. Due to the dual nature of H_2_O_2_ in the complete germination process, its concentration has to be strictly managed by antioxidant systems and remains between critical concentration thresholds called the “oxidative window” [23].

The level of lipid peroxidation caused by free radicals can be assessed by determining the concentration of thiobarbituric acid reactive substances (TBARS) such as malondialdehyde (MDA) [28]. Disruptions in lipid membranes affect their structure, increasing permeability [29] and causing electrolyte leakage, which is associated with viability loss [28,30]. Additionally, ultradry storage can strengthen the effect of oxidation by the dehydration of water film and exposure of molecule biofilms to free radicals present in the cell [31].

Ascobic acid (AsA) is a prevailing molecule that is present in different concentrations in vacuoles, cytosols, mitochondria and chloroplasts [32,33]. AsA acts as a reducer of free radicals, mainly H_2_O_2_, forming H_2_O and oxidizing AsA to nontoxic dehydroascorbate (DHA) via ascorbate peroxidase [34]. Subsequently, DHA is recycled to AsA by the oxidation of reduced glutathione (GSH). In plants, AsA mainly occurs in its reduced form in nonstress conditions, and the amount of oxidized forms of AsA increases when plant cells experience oxidative stress [35]. It is believed that AsA is present in high concentrations mainly in recalcitrant seeds and is almost absent in dry orthodox seeds [36,37]. AsA also affects extension biosynthesis [38], as well as root elongation, cell vacuolization and cell wall growth [32], acting in plant growth and development and their adaptation to environmental conditions. An oxidative intercellular environment results in increased redox potential values. Another indicator used for assessing the available AsA in cells is the AsA/DHA ratio [38].

The above data led us to perform a study of three basic markers (H_2_O_2_, TBARS and Ascrobate; Asc) in germinated seeds defined as orthodox exposed to oxidative stress under different seed storage conditions. The main aim of our study was to investigate how suboptimal storage conditions (moisture content <11% and temperature <-3°C) affect the germinated seeds at biochemical level, which conditions are most suitable for storing specific species seeds and how does it reflects further on seedling quality in three different species that produce orthodox seeds. Additionally, we wanted investigate if tested molecules could potentially be markers for seedling quality.

## Materials and methods

### Plant material

The study did not involve endangered or protected species. All seeds were collected from public and rural areas were no specific permissions were required. Mature seeds of the tested species, European crab apple (*Malus sylvestris* L.), wild cherry (*Prunus avium* L.) and bird cherry (*P*. *padus* L.), were collected in 2012 in natural stands in Poland. The seed lots of each species were collected from individual trees, cleaned and dried in ambient conditions. Seeds of each species were calibrated to three levels of approximate moisture content(MC), 5, 8 and 11% ([14]; Table 1), and stored in three-layered air-sealed polyethylene bags at temperatures of -3° C, -18°C or cryopreserved at -196°C for 24 and 36 months and stratified, as described in Wawrzyniak et al. [14]. The adjustment of the MC was based on the FW of the seeds. Seeds were desiccated in room temperature to ca. 11% and further under silica gel. Subsequently, seeds were placed in a moist substrate (a mixture of sand and peat) and underwent stratification [39]. Directly after stratification, germination and seedling emergence tests were conducted in a laboratory under controlled conditions according to the species requirements. Seeds with radicles >3 mm were considered germinated. Seeds with fully emerged cotyledons above the substrate were considered fully emerged. For both tests, we used three replicates of 30 seeds in each experimental treatment. The germination test was performed at a cyclically alternating temperature (3°/20°C, 16/8 h). Alternating temperature allows to finish the stratification procedure and to avoid the induction of secondary dormancy.

**Table 1:** Seedling emergence of *Malus sylvestris*, *Prunusavium* and *Prunuspadus* seeds (from Wawrzyniak et al. 2019) stored for two (2y) and three (3y) years and used as material in this report presenting the scheme of combination of storage temperature and moisture content (MC applied to seeds of each species);, SE–seedlings emergence.

Species	MC, %		Germination and seedlingemergence, %					
			2 years			3 years		
			Storage temperature, °C					
			-3	-18	-196	-3	-18	-196
*M*. *sylvestris*	4.9	SE	88.9	81.1	50.0	66.7	81.1	68.9
	8.5	SE	88.9	90.0	84.4	83.3	86.7	81.1
	10.7	SE	86.7	91.1	82.2	80.0	81.1	80.0
*P*. *avium*	5.5	SE	84.5	91.1	52.2	94.4	85.5	53.3
	8.0	SE	91.1	80.0	50.0	84.5	83.3	62.2
	11.2	SE	81.1	82.2	51.1	93.3	90.0	74.5
*P*. *padus*	5.9	SE	80.0	78.9	80.0	81.1	76.7	78.9
	8.3	SE	78.9	90.0	77.8	80.0	86.7	75.5
	11.4	SE	74.5	75.9	81.1	76.7	85.5	75.6

For biochemical analysis, we used germinated seeds (radicle > 3 mm) one week after release of dormancy. The biochemical samples consisted of five germinated seeds. For seedling analysis, we used ten leaves from three seedlings and three complete root systems produced after three months of growth in the growth chamber at 20°C, photoperiod: 16h day/8 night.

### Determination of hydrogen peroxide (H_2_O_2_) content

The samples (germinated whole seeds, leaves and roots of seedlings) were ground to a fine powder in liquid nitrogen. Next, they were homogenized with 5 mL of 5% TCA containing 10 mmol/L EDTA. The homogenate was centrifuged at 4°C at 12000 x g for 15 min. The total amount of supernatant was analyzed using the ferri-thiocyanate method according to Sagisaka [40].

### Estimation of lipid peroxidation (TBARS)

The lipid peroxidation level in seed tissues was determined by measuring the amount of 2-thiobarbituric acid reactive substances (TBARS) metabolites, mainly MDA, using the method of Heath and Packer [41]. This level was analyzed in the seeds of three species, *M*. *sylvestris*, *P*. *avium* and *P*. *padus*, that had been stored for two and three years under controlled MC and storage temperature conditions (Table 1). TBARS levels were also assessed in 3-month seedlings of the above species established from seeds stored for three years.

### Determination of ascorbate (Asc)

The AsA and DHA contents were assayed according to the method described by Kampfenkel et al. [42]. The samples in each of the three replicates were homogenized in cold 6% TCA (w/v) and centrifuged at 20000 x g for 20 min. The assay is based on the reduction of Fe^3+^ to Fe^2+^ by AsA in acidic solution. Fe^2+^ forms complexes with bipyridyl, giving a pink color with maximum absorbance at 525 nm. Total ascorbate was determined after the reduction of DHA to AsA by dithiothreitol. DHA in the assays was determined by subtracting the free AsA from the total AsA.

### Redox potential (E)

The half-cell reduction potential of ascorbate (E_DHA/AsA_) was calculated using the Nernst equation:

$$E=E_{0}-\frac{RT}{nF}\ln\frac{\left[Red\right]}{\left[Ox\right]}$$ (1)

where E_0_ is the standard half-cell reduction potential at pH 7 (E_0_ = 80 mV); R is the gas constant (8.314 JK^−1^ mol^−1^); T is the temperature [K]; n is the number of electrons involved in the reaction; F is the Faraday constant (96485.104C mol^−1^); Red is the molar concentration of reduced form (AsA); Ox is the molar concentration of the oxidized form (DHA).

### Statistical analyses

The presented data consist of means (SE) of three biological replicates. Statistical analysis of germinated seeds between means was performed using general linear model and included the main effects and interactions between seed moisture content, storage temperature and time (S1 Table). For pairwise comparisons between treatments, Tukey’s test was performed (p < 0.05) to verify whether the main effects were significant. Proportional data were transformed prior analysis using the arcsine transformation. As data collected from 3-month-old seedlings violated linear model criteria, comparisons between groups were performed using a nonparametric Kruskal-Wallis test followed by Dunn's post hoc test. For correlation, we used Pearson’s correlation. For all statistical analyses and visualizations of the data, R statistical software [43] was used. Correlation matrixes were made using the *corrplot* package [44].

## Results

### H_2_O_2_ levels

**Fig 1:**
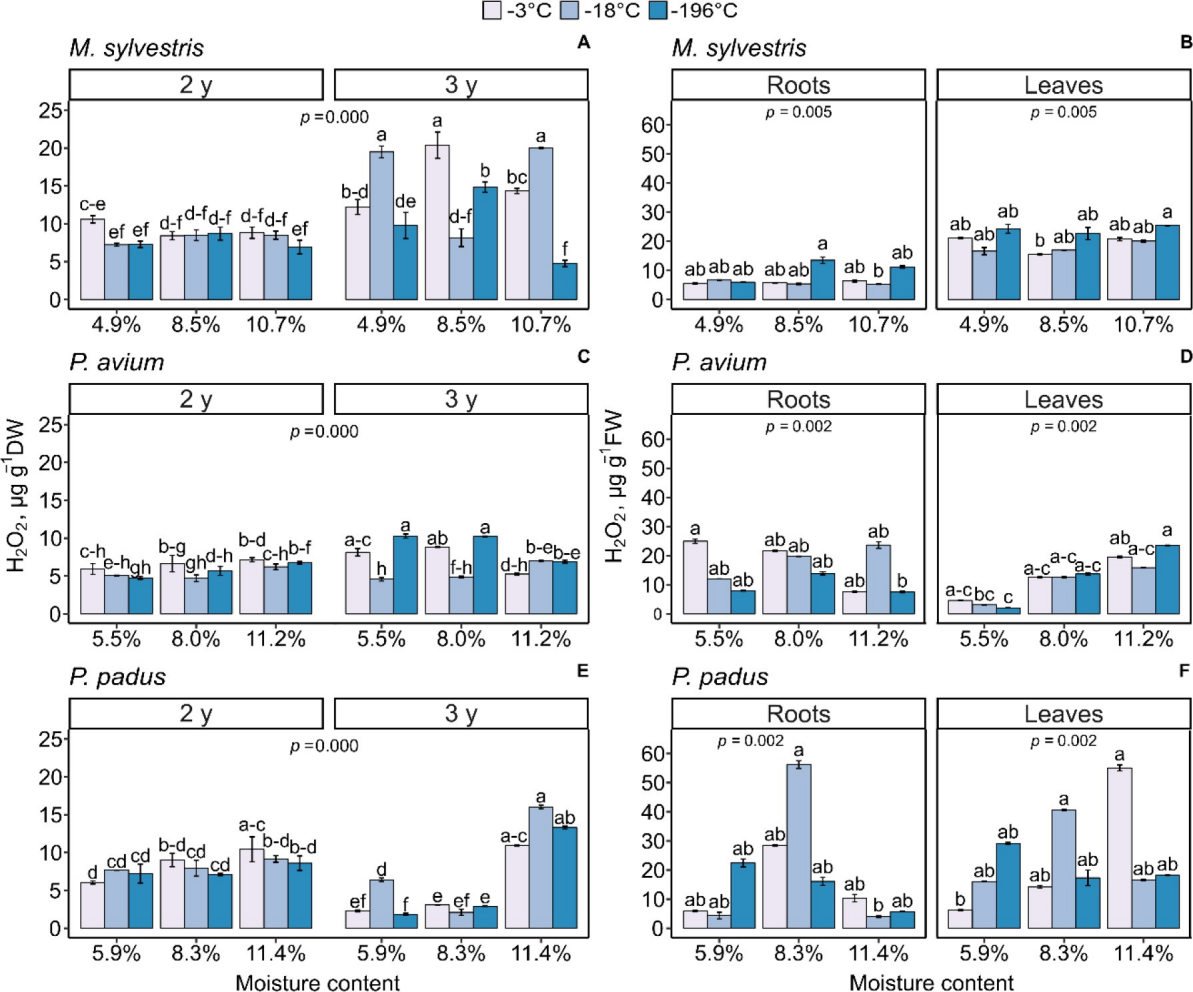
Changes in the levels of hydrogen peroxide (H_2_O_2_) reported in germianted seeds (A, C, E) and 3-month-old seedlings derived from the seeds (B, D, F) of *M*. *sylvestris* (A, B), *P*. *avium* (C, D) and *P*. *padus* (E, F). In germinated seeds, the level of H_2_O_2_ interaction between MC x storage temperature x storage time was compared. In 3-month seedlings, the level of H_2_O_2_ was measured in leaves and roots. Data are the means ± standard error obtained from three independent experiments with similar results. Statistically significant differences are indicated with different letters among the groups, followed by Tukey’s test for germinated seeds and Dunn’s test for seedlings at p ≤ 0.05.

The H_2_O_2_ levels were determined in both the germinated seeds and the seedlings derived from stored seeds (Fig 1). The level of H_2_O_2_ in *M*. *sylvestris* seeds after the 2^nd^ year of storage was similar in all tested combinations, except for the seeds stored at 4.9% MC. Seeds kept at -18°C and -196°C had a significantly lower level of H_2_O_2_ than those stored at -3°C (Fig 1A). After the third year of storage, H_2_O_2_ levels increased in almost all tested conditions, with the exception of seeds stored with 8.5% MC at a temperature of -3°C and 10.7% MC at a temperature of -196°C. The highest H_2_O_2_ level, which was reported in *M*. *sylvestris* seeds stored with 8.5% MC at a temperature of -3°C for three years, was two times greater than the H_2_O_2_ level of seeds stored under identical conditions for two years. Similarly, the H_2_O_2_ level doubled from year two to year three in germinated seeds stored with 4.9% and 10.7% MC at -18°C. The H_2_O_2_ level in the roots of *M*. *sylvestris* was half to that in the leaves of *M*. *sylvestris* in all tested conditions. There was a significant difference between the highest H_2_O_2_ content in roots of seedlings stored with 8.5% MC at -196°C and the lowest H_2_O_2_ content in seedlings stored with 10.7% MC at -18°C (Fig 1B). In the seedling leaves of all tested MC treatments, the highest H_2_O_2_ levels were found in leaves derived from seeds stored at -196°C. The leaves of seedlings established from seeds stored with 10.7% MC and at -196°C contained five times more H_2_O_2_ than the seeds from which they germinated.

In *P*. *avium* after two years of storage, there were no significant differences between the levels of H_2_O_2_ in all treatments. After the third year of storage, the H_2_O_2_ level increased in general but remained approximately constant when germinated seeds were stored at -18°C or with 11.2% MC. The highest level of H_2_O_2_ was reported for a temperature of -196°C with 5.5% and 8.0% seed MC (Fig 1C). In the roots of *P*. *avium* seedlings, the H_2_O_2_ level decreased in the 5.5% and 8.0% MC treatments as the storage temperature decreased from -3° to -196°C. The H_2_O_2_ level was the highest at 5.5% and -3°C, whereas the lowest level was detected in the 11.2% MC and -196°C treatment (Fig 1D). In leaves of 3-month seedlings, the overall level of H_2_O_2_ clearly increased with MC and was ten times higher in seeds stored with 11.2% MC compared to those stored with 5.5% MC. Extremely low H_2_O_2_ levels were characteristic of the leaves of seedlings established from seeds stored with 5.5% MC and at -196°C.

Germinated seeds of *P*. *padus* after two years of storage showed similar H_2_O_2_ levels in all tested treatments (Fig 1E). Furthermore, the average concentration of H_2_O_2_ was similar to that reported in *P*. *avium* seeds. Interestingly, the effect of seed MC on the H_2_O_2_ level appeared in the 3^rd^ year of storage when the level of H_2_O_2_ decreased in germinated seeds with 5.9% and 8.3% MC. In germinated seeds kept with 11.4% MC, the level of H_2_O_2_ increased and was clearly dependent on storage temperature. Both the roots and leaves of *P*. *padus* 3-month-old seedlings contained up to 20 times higher H_2_O_2_ concentrations than the seeds from which they were derived (Fig 1F). In both the roots and leaves of *P*. *padus* seedlings, extremely high levels of H_2_O_2_ were detected in seeds with 8.3% MC at -18°C. Inleaves, similarly high H_2_O_2_ levels were also reported in the 11.4% MC and -3°C treatment.

### TBARS levels

**Fig 2:**
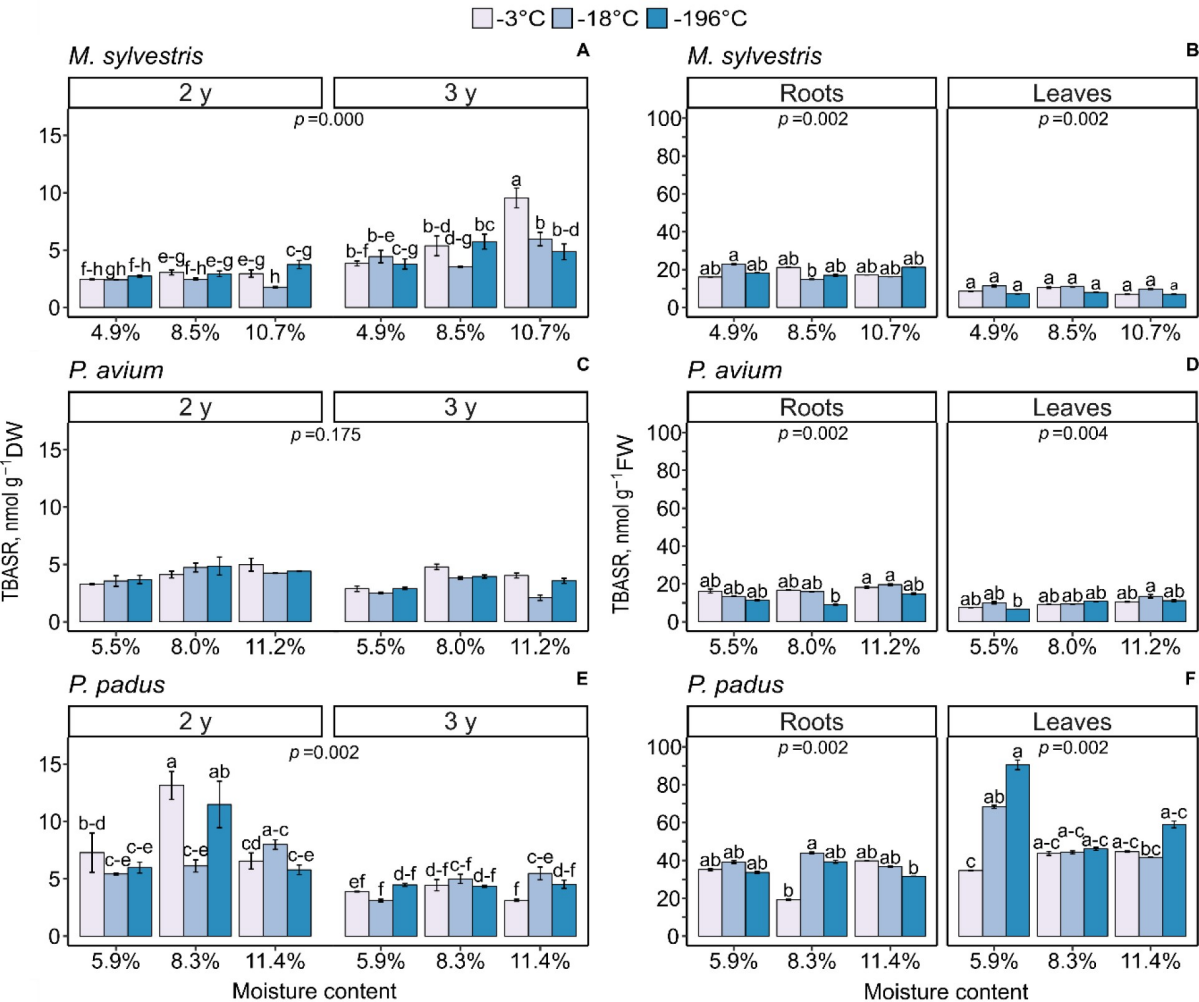
Changes in the levels of thiobarbituric acid reactive substances (TBARS) reported in germinated seeds (A, C, E) and 3-month-old seedlings derived from the seeds (B, D, F) *of M*. *sylvestris* (A, B), *P*. *avium* (C, D) and *P*. *padus* (E, F). In germinated seeds, the level of TBARS interaction between MC x storage temperature x storage time was compared. In 3-month seedlings, the level of TBARS was measured in leaves and roots. Data are the means ± standard error obtained from three independent experiments with similar results. Statistically significant differences are indicated with different letters among the groups, followed by Tukey’s test for germinated seeds and Dunn’s test for seedlings at p ≤ 0.05.

ROS degrade polyunsaturated lipids, causing lipid peroxidation and introducing lipid peroxidation end products, i.e., MDA. After two years of storage, germinated seeds of *M*. *sylvestris* contained a similar level of TBARS, approximately 3 nM g^-1^ DW, in all tested conditions except for seeds stored with 10.7% MC and at -18°C, which had the lowest TBARS values (Fig 2A). *M*. *sylvestris* seeds stored with 10.7% MC for two years showed the most diverse reaction with respect to TBARS production. In *M*. *sylvestris*, the level of TBARS increased in the germinated seeds stored for three years compared to the second year of seed storage. The TBARS levels were almost tripled in the germinated seeds stored with 10.7% MC at -3°C. Seeds dried to 4.9% MC and stored for three years contained identical and very low TBARS levels compared with seeds with higher MCs. The level of TBARS was significantly affected by the MC of seeds stored at -18°C for two and three years. In 3-month-old seedlings, the TBARS level was almost twice as high in roots than in leaves. TBARS levels were stabilized in leaves to approximately 9 nM g^-1^ FW in all tested treatments (Fig 2B). In roots, the average TBARS tripled compared to the initial level of TBARS in the seeds from which the seedlings originated.

In *P*. *avium* seeds stored for two years, the level of TBARS was similar among all treatments (Fig 2C). After the third year of storage, the TBARS level was significantly lower in germinated seeds stored with 5.5% MC in comparison to germinated seeds stored with 8.0% MC irrespective of the storage temperature. In *P*. *avium* seedlings, the level of TBARS was higher in roots than in leaves, and both were higher than the initial TBARS content in the germinated seeds from which the seedlings originated. In all tested MCs, the lowest level of TBARS was reported in seeds stored at -196°C, and the lowest values were observed in seedlings originating from seeds desiccated to 8.0% MC (Fig 2D). In leaves, TBARS levels were more unified among the different tested seed MCs.

The level of TBARS was the highest in *P*. *padus* germinated seeds stored with 8.3% MC at -3 and at -196°C for two years (Fig 2E). Other tested conditions did not affect the TBARS content. After the third year of storage, the TBARS concentration was similar and did not exceed 5 nM g^-1^ DW in all tested treatments. In contrast to other species analyzed, TBARS content in *P*. *padus* seedling roots was lower than in leaves when seeds stored with 5.9% MC were used for seedling establishment (Fig 2F). TBARS levels were approximately five times higher in roots than in germinated seeds. In general, the 5.9% MC treatment demonstrated the most diverse results, with TBARS levels clearly impacted by the seed storage temperature.

### Ascorbate levels

**Fig 3:**
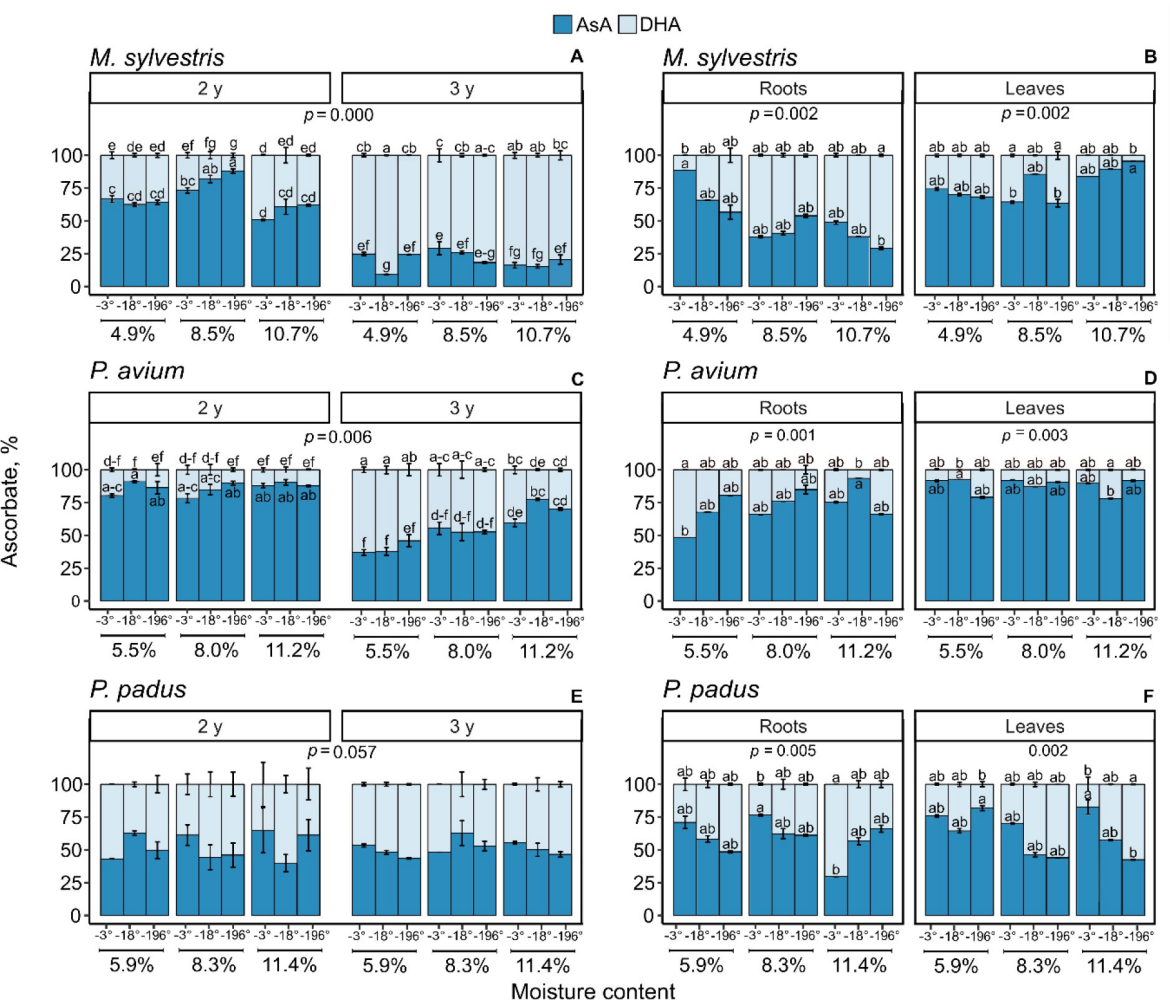
Changes in the reduced (AsA) and oxidized (DHA) form of ascorbate reported in germinated seeds (A, C, E) and 3-month-old seedlings derived from the seeds (B, D, F) of *M*. *sylvestris* (A, B), *P*. *avium* (C, D) and *P*. *padus* (E, F). In germinated seeds, the ratio of AsA and DHA between MC x storage temperature x storage time was compared. In 3-month seedlings, the ratio of ascorbate was measured in leaves and roots. Data are the means ± standard error obtained from three independent experiments with similar results. Statistically significant differences are indicated with different letters among the AsA and DHA (Tukey’s test for seeds and Dunn’s test for seedlings at p ≤ 0.05).

The ascorbate pool, which consisted of reduced and oxidized forms (Fig 3), as well as their precise concentrations, was investigated in the germinated seeds and 3-month seedlings of three species, *M*. *sylvestris*, *P*. *avium*, and *P*. *padus*.

Seeds of *M*. *sylvestris* stored for two years contained Asc mainly in a reduced form, comprising approximately 60% of the Asc pool when seeds were stored with 4.9 and 10.7% MC (Fig 3A). Interestingly, in the germinated seeds stored with 8.5% MC, the effect of storage temperature on Asc was observed. It was found that increasing AsA levels were reported for decreasing seed storage temperatures. After the third year of storage, the Asc pool halved and was dominated by the oxidized form of Asc. In general, the level of AsA did not exceed 25% of the total Asc. Extremely low AsA levels were detected in seeds stored at 4.9% MC and -18°C. Roots from *M*. *sylvestris* seedlings contained Asc concentrations similar to those of seeds stored for three years, whereas in leaves, the Asc concentrations were up to four times higher than those of seeds stored for three years. Roots from *M*. *sylvestris* seedlings contained less AsA and more DHA when seeds with 4.9 and 10.7% MC were stored at lower temperatures. AsA represented 88% and 29% of the total Asc pool in the most contrasting treatments. However, the concentration of Asc was higher in seeds with 10.7% MC than with 4.9% MC. The AsA and DHA levels in roots from seeds stored with 8.5% MC reached approximately 50% and remained constant irrespective of seed storage temperature. In *M*. *sylvestris* leaves, the majority of Asc was reduced, comprising more than 70% of the Asc pool for seeds stored with 4.9% and 8.5% MC, irrespective of the storage temperature. When seedlings were produced from seeds stored with higher MCs, the AsA level started to increase at decreasing storage temperatures and reached 95% of the total Asc pool when seeds were stored at -196°C.

Germinating seeds of *P*. *avium* stored for two years also contained Asc mainly in the reduced form (Fig 3C). AsA comprised at least 85% of the Asc pool. However, after two years of storage, the AsA and DHA concentrations of *P*. *avium* seeds were three times lower than those of *M*. *sylvestris* seeds. Similar AsA and DHA concentrations were reported in *P*. *avium* seeds stored for three years with 8.0% and 11.2% MC. Both AsA and DHA concentrations increased in seeds stored with 5.5% MC when the storage temperature decreased from -3° to -196°C (S1C Fig). The Asc concentration was doubled in seeds stored at -196°C in comparison to seeds stored at -3°C. After the third year of seed storage, the balance between the oxidized and reduced Asc forms varied and was clearly driven by the seed MC during storage. Independent of the storage temperature applied, AsA comprised an average of 40, 55, and 70% of the Asc pool in seeds stored at 5.5, 8.0, and 11.2% MC, respectively. The Asc levels in the roots and leaves of *P*. *avium* seedlings decreased by four and ten times, respectively, compared those in the seeds used for seedling establishment (Fig 3D). However, the AsA content was maintained in the 65–78% range. In the leaves of 3-month-old *P*. *avium* seedlings grown for three months, the Asc pool was dominated by AsA, which constituted an average of 88% of the Asc pool in each treatment (Fig 3D).

Germinated seeds of *P*. *padus* stored for two years maintained an average of 50% AsA and 50% DHA under each combination of storage conditions (Fig 3E). Additionally, after another year of storage, this redox balance was sustained in each treatment. Although the concentration of Asc was different in seeds stored for two years compared to seeds stored for three years. The percentage of the reduced Asc form was higher in the roots and leaves of *P*. *padus* seedlings than in the seeds used for seedling establishment (Fig 3F) and was dependent on decreasing storage temperature from -3° to -196°C. However, the Asc pool was more reduced for seeds with 5.9% MC than for germinated seeds with 8.5% MC. A clear storage temperature-dependent decrease in AsA levels was noticed in seeds with 11.4% MC, for which the AsA content changed from approximately 82% to 42%.The roots of seedlings established from seeds stored with 11.4% MC were characterized by an increasing AsA content, which was accompanied by a decreasing Asc concentration, mainly DHA, with decreasing storage temperature. In leaves, the AsA content was more unified in seeds stored with 5.9% and 8.5% MC. However, the Asc pool was more reduced for seeds with 5.9% MC than for seeds with 8.5% MC. A clear storage temperature-dependent decrease in AsA levels was noticed in seeds with 11.4% MC, for which the AsA content changed from approximately 82% to 42%.

### Redox potential

**Fig 4:**
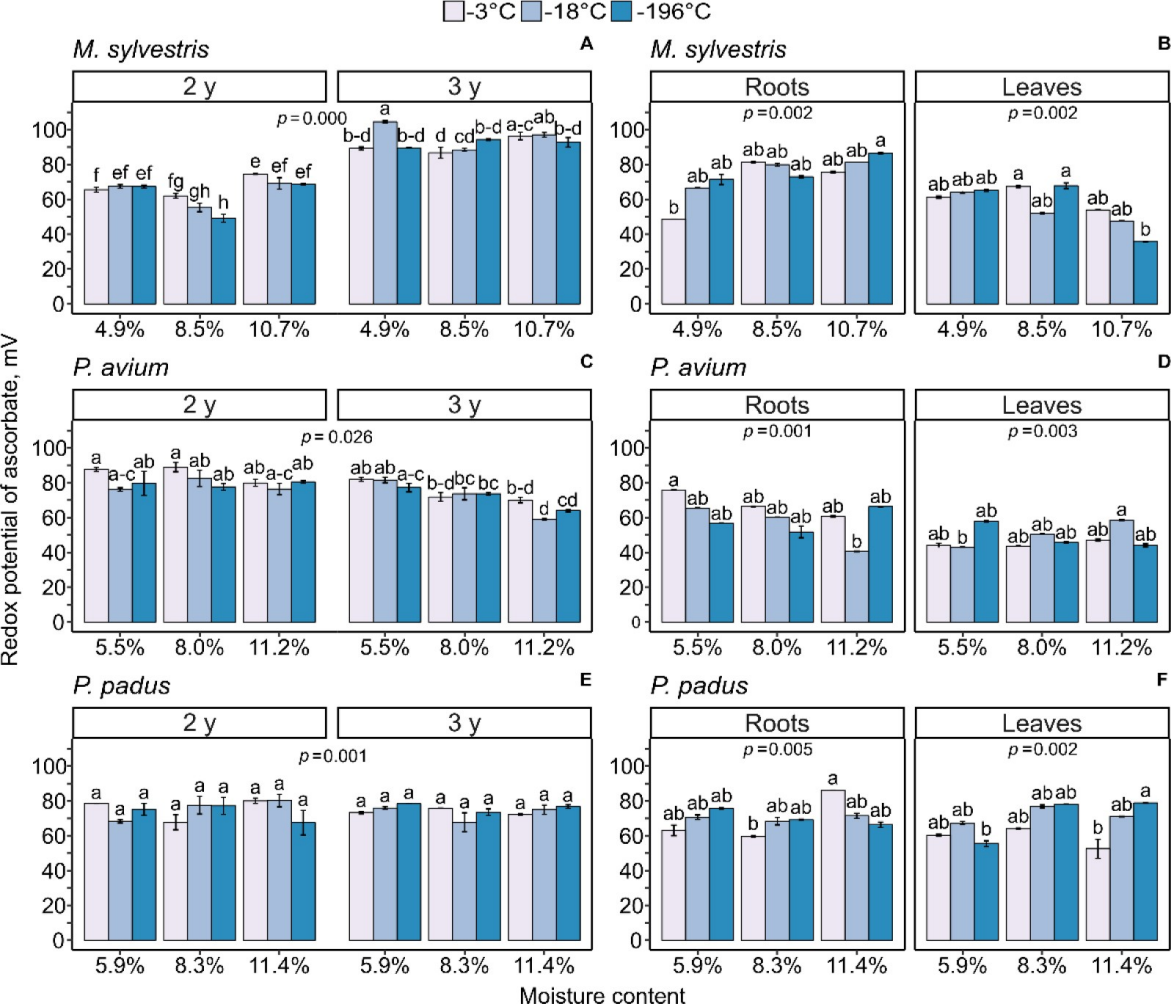
Changes in the levels of redox potential reported in germinated seeds (A, C, E) and 3-month-old seedlings derived from the seeds (B, D, F) of *M*. *sylvestris* (A, B), *P*. *avium* (C, D) and *P*. *padus* (E, F). In germinated seeds, redox potential level between MC x storage temperature x storage time was compared. In 3-month seedlings, the level of redox potential was measured in leaves and roots. Data are the means ± standard error obtained from three independent experiments with similar results. Statistically significant differences are indicated with different letters among the groups, followed by Tukey’s test for seeds and Dunn’s test for seedlings at p ≤ 0.05.

Levels of AsA and DHA determine the redox half-cell reduction potential (E_DHA/AsA_) in the following manner: the lower the E value is, the more reduced the Asc will be. E_DHA/AsA_ was calculated and compared in germinated seeds and seedlings derived from the three analyzed species (Fig 4). Among the seeds stored for two years, germinated seeds of *M*. *sylvestris* were characterized by the lowest E_DHA/AsA_ when seeds were stored with 8.5% MC (Fig 4A). After three years of storage the redox environment became more oxidized in germinated seeds of *M*. *sylvestris*, and the beneficial effect of seed storage with 8.5% MC was lost. In the roots of 3-month-old *M*. *sylvestris* seedlings, the E_DHA/AsA_ was 75 mV on average, except for two contrasting treatments representing statistically lower (4.9% MC at -3°C) and statistically higher (10.7% MC at -196°C) values of E_DHA/AsA,_ which differed by 42 mV (Fig 4B). The average E_DHA/AsA_ reported in leaves of *M*. *sylvestris* seedlings was more reduced than that in roots, except for the 10.7% MC treatment at a temperature of -196°C, in which the E_DHA/AsA_ was decreased to 35 mV, representing the most reduced environment observed in this report.

The E_DHA/AsA_was unified in germinated seeds of *P*. *avium* stored for two years irrespective of storage conditions. Interestingly, after three years of storage, a clear dependence on MC appeared (Fig 4C). The gradual increase in seed MC was accompanied by a gradual decrease in E_DHA/AsA_. In the roots of *P*. *avium* seedlings, the E_DHA/AsA_ was 60 mV on average, except for two contrasting treatments. differed by 35 mV (Fig 4D). E_DHA/AsA_ was more reduced in the leaves (approximately 48 mV) than in the roots of *P*. *avium* seedlings.

The AsA and DHA amounts were consistent after two years and three years of *P*.*padus*seed storage, as reflected in the unchanged E_DHA/AsA_, which remained at approximately 75 mV (Fig 4E). Furthermore, in *P*.*padus* seedlings, the E_DHA/AsA_reached approximately 70 mV in both roots and leaves (Fig 4F). There was a 27 mV difference between extremes in both organs.

### Correlation coefficient

Pearson’s correlation coefficient analysis between all examined parameters was performed (H_2_O_2_, TBARS, Asc, AsA, DHA, E, seedling emergence and germination) for seeds of each tested species stored for two and three years. In seeds of *M*. *sylvestris* stored for two years, concentrations of both AsA and Asc were negatively correlated with germination capacity and seedling emergence, as well as with t_50_, which indicates the time required to reach 50% of final germination capacity (S2A Fig). All these relationships were moderate (R between -0.40 and -0.55). After three years of storage, the AsA content changed and was positively correlated only with seedling emergence. With increasing concentrations of TBARS and AsA, the time required to reach 50% seed germination increased (S2B Fig).

The germination capacity of *P*. *avium* seeds after two years of storage was positively correlated with the redox potential (S3A Fig). However, in the case of the AsA:DHA ratio and AsA and Asc concentrations, a negative correlation was observed. After three years of storage, DHA and Asc concentrations were negatively correlated with germination capacity and the emergence of seedlings. In the same manner, the t_50_ values of seedling emergence and germination capacity were positively correlated with DHA and Asc concentrations, meaning that the time required for 50% of seeds to germinate increased with increasing concentrations of DHA and Asc (S3B Fig). Additionally, positive correlations between the H_2_O_2_ levels and t_50_ of both germination and emergence time were observed, and increasing germination time resulted in decreasing total germination capacity. Similarly, the AsA concentrations were negatively correlated with germination and seedling emergence and positively correlated with t_50_ germination (S3B Fig).

No significant correlation was found in *P*. *padus* seeds after 2 years of storage. After the third year, AsA concentrations and the AsA:DHA ratio were positively correlated with seedling emergence (S4A Fig), whereas the redox potential was negatively correlated with seedling emergence. All correlation strengths were moderate (S4A and S4B Fig).

In the roots of *M*. *sylvestris*, the concentration of DHA was positively correlated with seedling emergence (S2C Fig).

The level of TBARS in the roots of seedlings of *P*. *avium* and *P*. *padus* species significantly impacted the concentrations of at least one of the Asc forms. In the roots of *P*. *avium* seedlings, TBARS levels were correlated with the H_2_O_2_ level and AsA concentrations. The levels of TBARS also strongly affected seedling emergence. In addition to TBARS, seedling emergence was correlated with the level of H_2_O_2_, as well as the AsA, DHA and Asc concentrations, which was confirmed statistically (S3C Fig). In the leaves of *P*. *avium* seedlings, a strong positive correlation was reported between H_2_O_2_ and AsA concentrations; a similar correlation was also detected with Asc (S3D Fig).

The level of TBARS was positively correlated with AsA concentrations in both the roots and leaves of *P*. *padus* seedlings. In roots, the level of TBARS was additionally correlated with the DHA and Asc concentrations, E_DHA/AsA_ and AsA:DHA ratio (S4C and S4D Fig). In general, AsA concentration in germinating seeds stored for three years appeared to be the only one criterion correlated with seedling emergence in all three tested species (S5 Fig).

Comparison of data characterizing germinating seeds stored for two and three years clearly showed that storage conditions, i.e. temperature and moisture content, along with the extension of storage time significantly affect all parameteres measured in each species analyzed (S2 Table). Interestingly 52, 59 and 74% of tested combinations of storage conditions in three species were significantly affected levels of H_2_O_2_, lipid peroxidation and Asc, respectively (S2 Table) indicating that predominantly concentrations of AsA and DHA are affected by storage temperature and moisture content.

## Discussion

Seeds gradually age and lose their viability, as the seed life span is finite [1,2]. The life span of orthodox seeds is higher when stored dry and at low temperatures, but exceeding certain threshold increases seed deterioration and further ageing [45]. Thus, the beneficial effect of both drying and cooling on seed longevity still requires the search for an optimal moisture content–temperature combination that provides the maximum seed shelf life [13] because seed storage, including orthodox seeds, implicates seed deterioration [46] thereby affecting successful seedling performance. A high seedling quality is vital in the survival in nature and withstanding environmental stress, both of abiotic and biotic origin. [47,48]. Therefore effects of seed storage on early seedling establishment need to be understood to produce high-quality planting stock. However, studies researching seedling physiology after seeds storage are still scarce in the literature, they confirm a possible effect on seedling quality [49–51]. The effect of storage conditions on seedling performance is here discussed in terms of searching early signs of deterioration in stored orthodox seeds.

### Oxidation processes in stored seeds

The dual role of ROS in seed physiology depends on ROS concentrations, which determine whether ROS function as signaling molecules or, in contrast, lead to oxidative stress [52]. Sies [53] determined that H_2_O_2_ effects are dose-specific and that H_2_O_2_ plays a signaling role in the range of 1–10 nM concentrations perceived by plants as oxidative eustress. In contrast, oxidative distress introduces oxidative damage [54]. H_2_O_2_, considered as signaling molecule, is involved in regulation of seed germination process [56–58], and is further essential to the normal growth and development of seedlings [55]. Thus, steady-state H_2_O_2_ concentrations need to be characterized to determine the limits of the H_2_O_2_ levels differentiating eustress from distress ranges in seeds. Decreased germination capacity linked with increased H_2_O_2_ and lipid peroxidation levels was reported in stored tree seeds representing the intermediate category such as beech seeds [59] and Poplar seeds [22]. Importantly, storage conditions, including temperature and MC, affected ROS production in the seeds of both species [23,52]. The above data support the necessity of estimating optimal storage conditions separately for species that can differ considerably within the orthodox category and validate the applicability of our study.

Because many forms of damage, including ROS-derived damage, serve as fundamental factors determining seed deterioration and further seed aging, the prototypical free radical theory of aging was replaced by a more accurate model that considers biological imperfectness as the true cause of aging [60]. Recently, Ren and Zhang [61] suggested that aging is encoded by genes or DNA, and pro-aging factors accelerate and promote this process in contrast to anti-aging factors that retard this process. H_2_O_2_ is considered to be a pro-aging factor [21]. As shown in this report, H_2_O_2_ levels significantly affected germination capacity and seedling emergence uniquely in *P*. *avium* (Table 1), which suggests the occurrence of oxidative stress. The impact of H_2_O_2_ levels on TBARS levels and the AsA:DHA ratio was prominent in the roots of *M*. *sylvestris* seedlings and resulted in the selection of oxidation-introducing parameters affecting seedling emergence. The controlled deterioration treatment of elm seeds resulted in doubled H_2_O_2_ levels [62]. Some treatments doubled H_2_O_2_ levels in germinated *M*. *sylvestris* seeds stored for three years emphasizing stress symptoms. However, in the majority of seeds, doubled H_2_O_2_ levels did not overlap with significantly increased TBARS levels, allowing us to hypothesize that lipid peroxidation is not the result of accumulated H_2_O_2_ but more likely lipid auto-oxidation is the origin of increased membrane permeability. Particularly, auto-oxidation is intensified in seeds dried below 6% MC [63].

The increase in TBARS levels was clearly reported in specific treatments of germinated *M*. *sylvestris* seeds. More spectacular changes in MDA levels were observed when seed viability decreased to approximately 60%. The lowest germination capacity (69% on average) occurred in *M*. *sylvestris* seeds stored for three years with 5% MC [14] notably, in these seeds, TBARS levels increased 1.6 times (Fig 2B). MDA significantly increased in orthodox-type willow seeds stored for 16 years, whereas seeds stored for up to 10 years were characterized by unaffected MDA levels [64]. In contrast, MDA levels increased by several dozen times during the accelerated aging of pepper seeds [65]. MDA, the main component of TBARS, is undoubtedly well-established marker of oxidative stress. The majority of methods enable measuring only free MDA omitting MDA-conjugates, thus not reflecting the total amount of MDA generated from lipid peroxidation [66] and rendering distinct results and their explanations.

Ebone et al. [46] proposed three phasic deterioration was described in stored orthodox seeds. A decline in protective mechanisms against oxidative damages is observed in phase I, membrane damage followed by lipid peroxidation appears in phase II, viability reduction and eventually inhibition of germination is characteristic to phase III. Based on the Ebone et al. [46] classification, all three species experienced phase I of seed deterioration. Seeds entered phase II of deterioration only in certain combination of storage treatments resulting in evidently increased lipid peroxidation reported in *M*. *sylvestris* or disrupted *P*. *avium* seedling emergence. Viable seedlings with suppressed growth are characteristic to phase II of deterioration [67] and significantly lower *P*. *avium* seedlings were produced from seeds stored at -196°C [14]. Prolonged seed storage increases the redox potential for both glutathione (E_GSSG/2GSH_) and ascorbate (E_DHA/AsA_) in different genotypes and can be used to monitor seed viability [23,68]. Principally in *M*. *sylvestris* and *P*. *avium* seeds the E_DHA/AsA_ increased during storage supporting our hypothesis that the two species display more advanced symptoms of deterioration as compared to *P*. *padus*. Surprisingly, our investigation showed that lower TBARS levels were detected in *P*. *padus* seeds stored for three years than in those stored for two years. A similar phenomenon was reported in artificially aged *P*. *sativum* seeds after 25 d; however, after 55 d, TBARS levels declined and returned to the levels observed before the aging treatment [69]. Olivier et al. [70] demonstrated that the minimization of damage in living tissues under desiccation and the activation of mechanisms and repair systems during hydration enable orthodox seed survival in a viable condition. Comparing seeds of three species after three years of storage, *P*. *padus* seeds seem to be exceptionally balanced. Probably, *P*. *padus* seeds have a broad range of tolerance to storage conditions such as MC and temperature, which was confirmed by Popova et al. [71], as *P*. *padus* seeds can withstand desiccation to 3.5%.

### Can Ascorbate be the early marker of seed deterioration and seedling quality?

Importantly, the parameters that significantly affected seedling emergence in all three species were the AsA and DHA levels, AsA/DHA ratio, and E_DHA/AsA_. AsA levels were linked to germination capacity and seedling emergence of *M*. *sylvestris* and *P*. *avium* (S1 and S2 Figs).*P*. *padus* possibly applied a different strategy of redox control by maintaining a stable 1:1 ratio of AsA to DHA in seeds (Fig 3E) resulting in successful seedling emergence. Redox changes of water-soluble antioxidants, including AsA, are regarded as seed viability markers [72]. Particularly, in *M*. *sylvestris* seeds, the Asc concentrations decreased 2.5–8.5 times depending on combination of storage conditions. Asc is sensitive to storage temperature, storage time and moisture content, however the scenario of the combined effect of moisture and temperature on Asc stability is still unknown [73]. Depletion in AsA levels might be the reason why germination capacity of *M*. *sylvestris* seeds was statistically lower in seeds stored with 4.9% MC than in seeds stored at other MCs because AsA was assumed to be a key antioxidant forcing viability from orthodox [68] through intermediate [74] up to recalcitrant [75,76] seeds. A low Asc pool level, in which DHA dominates, is necessary for the maintenance of the root quiescent center [77], thereby indicating that AsA oxidation should be considered in the context of the regulation of root growth and development. DHA constituted 26% of the Asc pool reported in the roots of *P*. *avium*, whereas the roots of *P*. *padus* contained 41% DHA, suggesting more suitable conditions for development of in *P*. *padus* roots.

Based on concentrations of Asc in seedlings leaves of *M*. *sylvestris* synthesized twice more Asc than *P*. *avium* leaves and nearly four times more than *P*. *padus* leaves (S1 Fig). At further growth stages Asc distribution differs across canopy profile reaching higher concentrations in leaves of the top and middle canopy layers [78]. Higher Asc levels reported in *M*. *sylvestris* would possibly promote protection of the phosphosynthetic apparatus because Asc-based photoprotection is well documented in woody plants (reviewed in Bilska et al. [79]. Growth abnormalities were reported in plants displaying decreased Asc levels and deficiency in AsA to DHA switches [80,81]. In this context, seedlings displaying higher Asc levels in leaves might be highly competitive in growth as compared to other species. Apart from interspecies differences in the levels of Asc which were reported even in seedlings of the same *Quercus* genus [82], higher endogenous Asc levels would definitely benefit seedlings under high light, drought, heat, cold, air pollution, acid rain and biotic infections [79]. Thus, hypothetically, *M*. *sylvestris* seedlings are better equipped to win the competition with seedlings of *P*. *avium* land *P*. *padus* when growing under identical environmental conditions. Interestingly, concentrations of DHA in leaves were correlated with seedling emergence solely in *M*. *sylvestris* supporting the above hypotheses.

Remarkably, the Asc not only responsible for the maintenance of seed viability during storage, but along with ROS participates in the modulation of gene expression that activate the suitable hormones responsible for the proper growth and development of the seedling [83,84] and adaptation to the prevailing environmental conditions [85].

### The difficulty in selection optimal storage conditions

The principles and practices of seed storage involve the preservation of seeds under controlled environmental conditions, ensuring their viability and further conservation of species and ecosystem biodiversity. Orthodox seeds are usually stored at subzero temperatures in gene banks. For ex situ conservation of orthodox seeds, a storage temperature of -18°C is recommended in general [86]. However, for long-term storage, cryopreservation methods are also used for orthodox seeds, both as a backup or for necessity [1]. For some forest trees, such as *P*. *avium*[15], *P*. *padus* [71], and *M*. *sylvestris* [87], cryopreservation is an established methodology.

All three species analyzed in this report produce seeds classified as orthodox; however, they differ in responses to storage conditions and the intensification of oxidative processes accompanying aging and seedling establishment. *P*. *avium* seeds are considerably sensitive to oxidation events during seed storage. Based on our results, progressive oxidation distress can be expected during *P*. *avium* seed storage, followed by continuous aging, making seeds of this species especially vulnerable to viability loss. In contrast, *P*. *padus* seeds seem to be extremely balanced in terms of oxidative processes under all applied storage conditions, at least in the first three years of storage. In the case of *M*. *sylvestris* seeds, severe desiccation should be avoided until the cause of its decreased seedling establishment is identified because seedling emergence was the lowest in seeds with 4.9% MC. Such a decrease in seedling emergence in severely desiccated seeds of *M*. *sylvestris* was reported by Michalak et al. [87] and Wawrzyniak et al. [14].

Based on both the seed germination and seedling examination results of this report, the most favorable storage conditions enabling low levels of H_2_O_2_, TBARS and AsA, indicating eustress in seeds, were as follows: 10.7% MC and -196°C for *M*. *sylvestris* seeds, 5.5% MC and -18°C for *P*. *avium* seeds and 5.3% MC and -3°C for *P*. *padus* seeds. Considering that the above parameters are not the only parameters determining the seed germination capacity, additional analyses are required to establish the ideal storage protocol for each species; however, this study clearly indicates that after three years of storage, oxidative distress occurs under the selected storage conditions.

## Conclusions

We proved that the AsA concentrations observed in seeds stored for three years significantly affected seedling emergence in all three species, emphasizing that, AsA is a reliable seed viability marker important in maintaining a specific redox environment. The depletion of the Asc pool is likely one of the reasons for the poor seedling performance of cryostored *M*. *sylvestris* seeds previously dried to 4.9% MC. H_2_O_2_ levels significantly affected germination capacity uniquely in *P*. *avium* seeds stored for three years. Additionally, *P*. *avium* was the only species in which seedling emergence was clearly determined by the synergistic action of the three tested oxidation markers, H_2_O_2_, TBARS and AsA levels. Remarkably, *P*. *padus* seeds had a broad range of tolerance to the tested MCs and storage temperatures, resulting in balanced reduction and oxidation processes enabling a high germination capacity and successful seedling establishment. Distinct molecular responses of species, from the same category (orthodox seeds) and from the same niche after different seed storage, occurring during seed germination and establishment of healthy seedlings, emphasize differences in seed resilience concerning feasibility of their storage in ultra-low temperature and seed moisture content.

## Supporting information

S1 Table General linear model analysis summary in germinated seeds for all tested molecules. Df- degree of freedom; Pr–probability; Chi–chi-square.
(DOCX)

S2 Table ANOVA analasis of biochemical markers after second and third year of storage. Upper arrow shows significant increase in measured marker. Down arrow indicates significant decrease.
(DOCX)

S1 Fig Reduced (AsA) and oxidized (DHA) forms of ascorbate in stored seeds of M. sylvestris, P. avium and P. padus. Seeds were stored at three moisture contents ca. 5, 8, 11% and three temperatures -3°, -18°, -196°C for two and three years. Mean ± s.e. Different letters indicate significant differences among the groups (Dunn’s test for. P < 0.05).
(DOCX)

S2 Fig Correlation matrices for seeds of *M*. *sylvestris* stored for two years (A), three years (B), roots (C) and leaves (D) of 3-month old seedlings. Crossed numbers indicate non-significant correlation (P < 0.05).
(DOCX)

S3 Fig Correlation matrices for seeds of *P*. *avium* stored for two years (A), three years (B), roots (C) and leaves (D) of 3-month old seedlings. Crossed numbers indicate non-significant correlation (P < 0.05).
(DOCX)

S4 Fig Correlation matrices for seeds of *P*. *padus* stored for two years (A), three years (B), roots (C) and leaves (D) of 3-month old seedlings. Crossed numbers indicate non-significant correlation (P < 0.05).
(DOCX)

S5 Fig Correlation between seedling emergence and AsA content of *M*. *sylvestris*, *P*. *avium* and *P*. *padus* seeds after three years storage in different temperature. (DOCX)
