# Supplementary material for: Oxidation processes related to seed storage and seedling growth of Malus sylvestris, Prunus avium and Prunus padus
Source: PLoS One. 2020 Jun 18;15(6):e0234510. doi: 10.1371/journal.pone.0234510 (PMC7302524; doi:10.1371/journal.pone.0234510)
Supplement: S1 Table — Df- degree of freedom; Pr–probability; Chi–chi-square. (DOCX) [file pone.0234510.s001.docx]

**S1 Table. General Linear Model analysis summary in germinated seeds for all tested molecules.** Df- degree of freedom; Pr – probability; Chi – chi-square.

| **Species** | **Source** | **Df** | **Explained Deviance** | **Residual Deviance** | **% Explained** | **Pr(>Chi)** | **Df** | **Explained Deviance** | **Residual Deviance** | **% Explained** | **Pr(>Chi)** |
| --- | --- | --- | --- | --- | --- | --- | --- | --- | --- | --- | --- |
| **Responce : H_2_O_2_** | | | | | | | **Responce : MDA** | | | | |
| *M. sylvestris* | Null |  |  | 1252.48 |  |  |  |  | 9.2485 |  |  |
|  | MC | 2 | 8.15 | 1244.33 | 0.65 | 0.160 | 2 | 0.6589 | 8.5895 | 7.12 | **0.000***** |
|  | T | 2 | 149.54 | 1094.80 | 11.94 | **0.000***** | 2 | 0.5770 | 8.0125 | 6.24 | **0.000***** |
|  | ST | 1 | 399.34 | 695.46 | 31.88 | **0.000***** | 1 | 5.1335 | 2.8790 | 55.51 | **0.000***** |
|  | MC x T | 4 | 254.19 | 441.27 | 20.29 | **0.000***** | 4 | 0.5155 | 2.3635 | 5.57 | **0.000***** |
|  | MC x ST | 2 | 2.16 | 439.11 | 0.17 | 0.616 | 2 | 0.4675 | 1.8960 | 5.05 | **0.000***** |
|  | T x ST | 2 | 77.77 | 361.34 | 6.21 | **0.000***** | 2 | 0.2915 | 1.6045 | 3.15 | 0.003** |
|  | MC x T x ST | 4 | 281.37 | 79.98 | 22.47 | **0.000***** | 4 | 0.7190 | 0.8855 | 7.77 | **0.000***** |
| *P. avium* | Null |  |  | 3.8654 |  |  |  |  | 3.5820 |  |  |
|  | MC | 2 | 0.03772 | 3.8277 | 0.98 | 0.252 | 2 | 1.01317 | 2.5688 | 28.29 | **0.000***** |
|  | T | 2 | 0.88782 | 2.9399 | 22.97 | **0.000***** | 2 | 0.26883 | 2.3000 | 7.51 | **0.000***** |
|  | ST | 1 | 0.52829 | 2.4116 | 13.67 | **0.000***** | 1 | 0.71373 | 1.5863 | 19.93 | **0.000***** |
|  | MC x T | 4 | 0.53338 | 1.8782 | 13.8 | **0.000***** | 4 | 0.28681 | 1.2994 | 8.01 | **0.005**** |
|  | MC x ST | 2 | 0.43059 | 1.4476 | 11.14 | **0.000***** | 2 | 0.20075 | 1.0987 | 5.60 | **0.005**** |
|  | T x ST | 2 | 0.49376 | 0.9538 | 12.77 | **0.000***** | 2 | 0.28795 | 0.8107 | 8.04 | **0.000***** |
|  | MC x T x ST | 4 | 0.46107 | 0.4928 | 11.93 | **0.000***** | 4 | 0.12133 | 0.6894 | 3.39 | 0.175 |
| *P. padus* | Null |  |  | 22.9993 |  |  |  |  | 8.3805 |  |  |
|  | MC | 2 | 9.4793 | 13.5200 | 41.22 | **0.000***** | 2 | 0.9818 | 7.3987 | 11.72 | **0.000***** |
|  | T | 2 | 0.4812 | 13.0388 | 2.09 | **0.000***** | 2 | 0.0488 | 7.3499 | 0.58 | 0.442 |
|  | ST | 1 | 0.4812 | 9.3328 | 2.09 | **0.000***** | 1 | 4.1655 | 3.1844 | 49.70 | **0.000***** |
|  | MC x T | 4 | 1.5435 | 7.7893 | 6.71 | **0.000***** | 4 | 0.9355 | 2.2489 | 11.16 | **0.000***** |
|  | MC x ST | 2 | 5.4410 | 2.3483 | 23.66 | **0.000***** | 2 | 0.2377 | 2.0112 | 2.84 | **0.018*** |
|  | T x ST | 2 | 0.2807 | 2.0676 | 1.22 | **0.005 **** | 2 | 0.4132 | 1.5980 | 4.93 | **0.001**** |
|  | MC x T x ST | 4 | 1.1320 | 0.9356 | 4.92 | **0.000***** | 4 | 0.5213 | 1.0767 | 6.22 | **0.002**** |

| **Species** | | **Source** | **Df** | | **Explained Deviance** | | **Residual Deviance** | | **% Explained** | **Pr(>Chi)** | **Df** | **Explained Deviance** | **Residual Deviance** | **% Explained** | **Pr(>Chi)** |  |
| --- | --- | --- | --- | --- | --- | --- | --- | --- | --- | --- | --- | --- | --- | --- | --- | --- |
| **Responce : Asc** | | | | | | | | | | | **Responce : Redox** | | | | | |
| *M. sylvestris* | Null | |  |  | | 13758.1 | |  | |  |  |  | 13771.0 |  |  |  |
|  | MC | | 2 | 968.6 | | 12789.5 | | 7.04 | | **0.000***** | 2 | 1070.0 | 12701.0 | 7.77 | **0.000***** |  |
|  | T | | 2 | 73.9 | | 12715.6 | | 0.54 | | **0.009**** | 2 | 104.1 | 12596.9 | 0.76 | **0.003**** |  |
|  | ST | | 1 | 11596.6 | | 1119.0 | | 84.29 | | **0.000***** | 1 | 11251.7 | 1345.1 | 81.71 | **0.000***** |  |
|  | MC x T | | 4 | 207.4 | | 911.6 | | 1.51 | | **0.000***** | 4 | 254.1 | 1091.0 | 1.85 | **0.000***** |  |
|  | MC x ST | | 2 | 239.6 | | 672.1 | | 1.74 | | **0.000***** | 2 | 224.2 | 866.8 | 1.63 | **0.000***** |  |
|  | T x ST | | 2 | 169.5 | | 502.6 | | 1.23 | | **0.000***** | 2 | 213.4 | 653.4 | 1.55 | **0.000***** |  |
|  | MC x T x ST | | 4 | 220.0 | | 82.5 | | 1.60 | | **0.000***** | 4 | 331.9 | 321.5 | 2.41 | **0.000***** |  |
| *P. avium* | Null | |  |  | | 8407.4 | |  | |  |  |  | 3624.8 |  |  |  |
|  | MC | | 2 | 848.4 | | 7559.0 | | 10.09 | | **0.000***** | 2 | 772.32 | 2852.5 | 21.31 | **0.000***** |  |
|  | T | | 2 | 211.3 | | 7347.8 | | 2.51 | | **0.000***** | 2 | 290.88 | 2561.6 | 8.02 | **0.001**** |  |
|  | ST | | 1 | 6029.8 | | 1317.9 | | 71.72 | | **0.000***** | 1 | 967.30 | 1594.3 | 26.69 | **0.000***** |  |
|  | MC x T | | 4 | 66.3 | | 1251.6 | | 0.79 | | 0.332 | 4 | 90.46 | 1503.9 | 2.50 | 0.387 |  |
|  | MC x ST | | 2 | 513.7 | | 737.9 | | 6.11 | | **0.000***** | 2 | 435.22 | 1068.6 | 12.01 | **0.000***** |  |
|  | T x ST | | 2 | 9.8 | | 728.1 | | 0.12 | | 0.712 | 2 | 40.23 | 1028.4 | 1.11 | 0.398 |  |
|  | MC x T x ST | | 4 | 208.0 | | 520.0 | | 2.47 | | **0.006**** | 4 | 241.76 | 786.6 | 6.67 | **0.026*** |  |
| *P. padus* | Null | |  |  | | 3265.1 | |  | |  |  |  | 2061.1 |  |  |  |
|  | MC | | 2 | 40.7 | | 3224.4 | | 1.25 | | 0.70419 | 2 | 41.38 | 2019.7 | 2.01 | 0.519 |  |
|  | T | | 2 | 76.98 | | 3147.4 | | 2.36 | | 0.515 | 2 | 3.92 | 2015.8 | 0.19 | 0.940 |  |
|  | ST | | 1 | 13.59 | | 3133.8 | | 0.42 | | 0.628 | 1 | 2.41 | 2013.4 | 0.12 | 0.782 |  |
|  | MC x T | | 4 | 332.60 | | 2801.2 | | 10.19 | | 0.220 | 4 | 208.57 | 1804.8 | 10.12 | 0.158 |  |
|  | MC x ST | | 2 | 86.19 | | 2715.0 | | 2.64 | | 0.476 | 2 | 35.23 | 1769.6 | 1.71 | 0.572 |  |
|  | T x ST | | 2 | 100.08 | | 2614.9 | | 3.07 | | 0.422 | 2 | 76.60 | 1693.0 | 3.72 | 0.297 |  |
|  | MC x T x ST | | 4 | 525.95 | | 2089.0 | | 16.11 | | 0.06 | 4 | 556.44 | 1136.5 | 27.00 | **0.001**** |  |
