# Supplementary material for: Oxidation processes related to seed storage and seedling growth of Malus sylvestris, Prunus avium and Prunus padus
Source: PLoS One. 2020 Jun 18;15(6):e0234510. doi: 10.1371/journal.pone.0234510 (PMC7302524; doi:10.1371/journal.pone.0234510)

S2 Table. ANOVA analasis of biochemical markers after second and third year of storage. Upper arrow shows significant increase in measured marker. Down arrow indicates significant decrease.
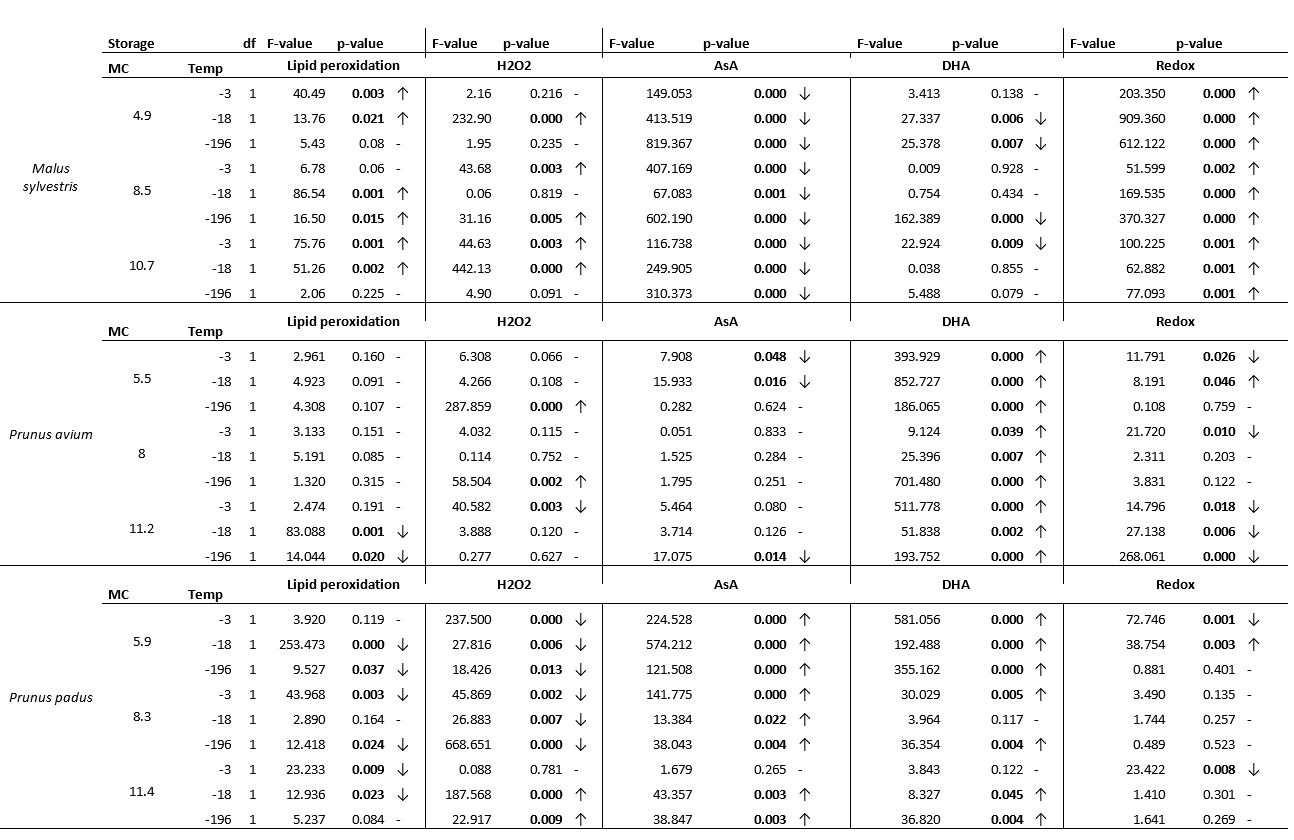

Supplement: S2 Table — Upper arrow shows significant increase in measured marker. Down arrow indicates significant decrease. (DOCX) [file pone.0234510.s002.docx]
