# Supplementary material for: Oxidation processes related to seed storage and seedling growth of Malus sylvestris, Prunus avium and Prunus padus
Source: PLoS One. 2020 Jun 18;15(6):e0234510. doi: 10.1371/journal.pone.0234510 (PMC7302524; doi:10.1371/journal.pone.0234510)
Supplement: S1 Fig — Seeds were stored at three moisture contents ca. 5, 8, 11% and three temperatures -3°, -18°, -196°C for two and three years. Mean ± s.e. Different letters indicate significant differences among the groups (Dunn’s test for. P < 0.05). (DOCX) [file pone.0234510.s003.docx]

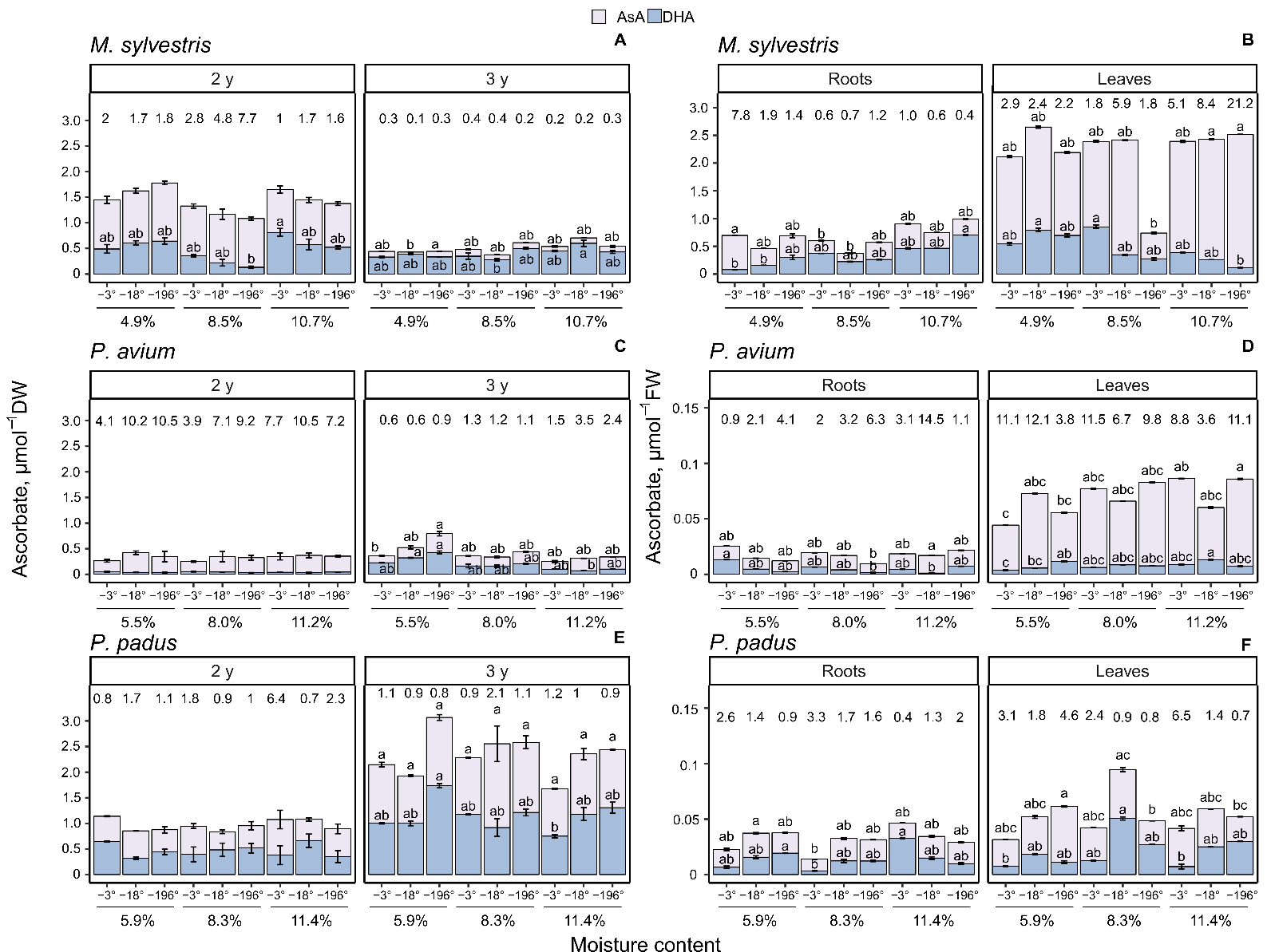


**S1 Fig.** Reduced (AsA) and oxidized (DHA) forms of ascorbate in stored seeds of *M. sylvestris*, *P. avium* and *P. padus*. Seeds were stored at three moisture contents ca. 5, 8, 11% and three temperatures -3°, -18°, -196°C for two and three years. Mean ± s.e. Different letters indicate significant differences among the groups (Dunn’s test for. P < 0.05).
