# Supplementary material for: Oxidation processes related to seed storage and seedling growth of Malus sylvestris, Prunus avium and Prunus padus
Source: PLoS One. 2020 Jun 18;15(6):e0234510. doi: 10.1371/journal.pone.0234510 (PMC7302524; doi:10.1371/journal.pone.0234510)
Supplement: S4 Fig — Correlation matrices for seeds of P. padus stored for two years (A), three years (B), roots (C) and leaves (D) of 3-month old seedlings. Crossed numbers indicate non-significant correlation (P < 0.05). (DOCX) [file pone.0234510.s006.docx]

***P. padus***


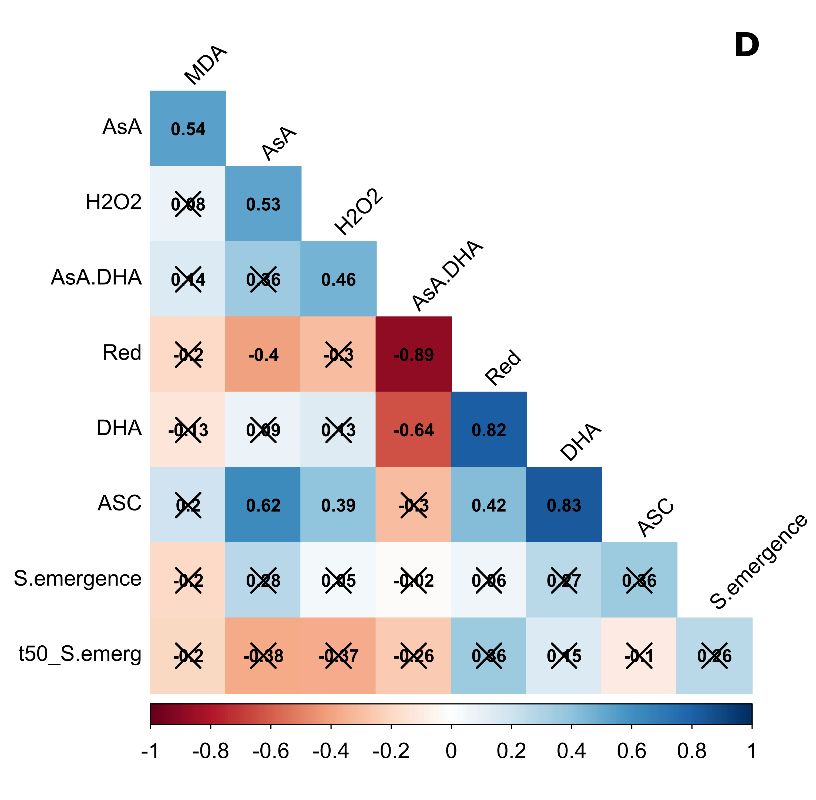

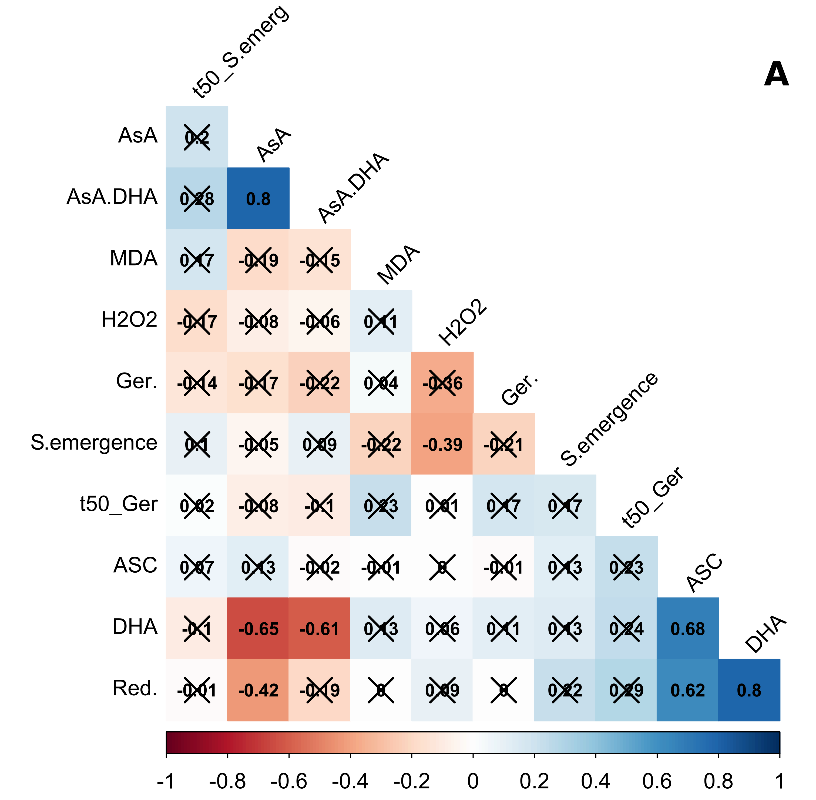

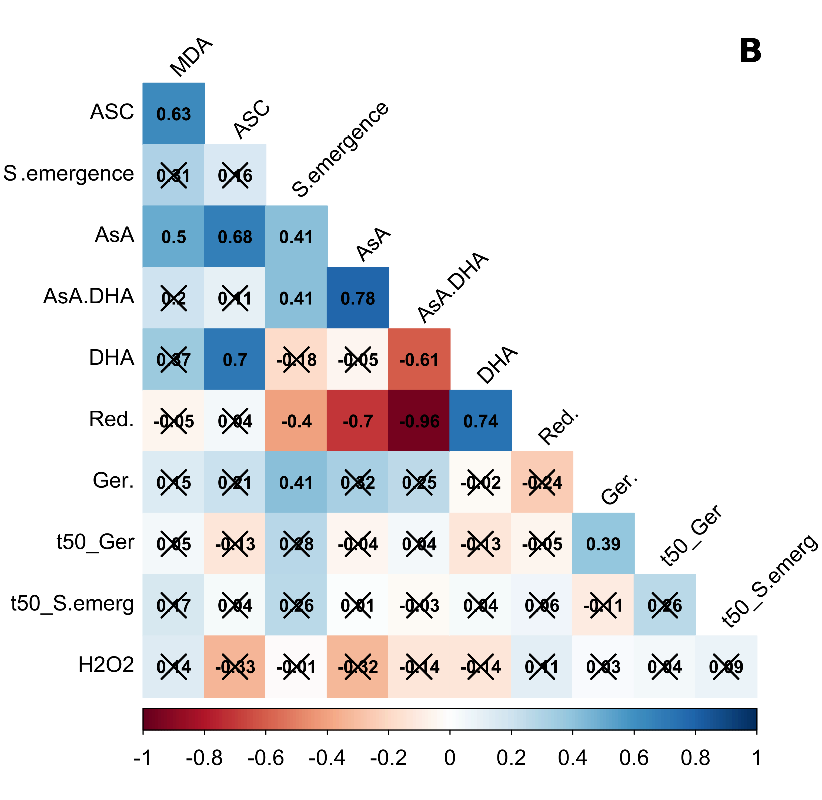

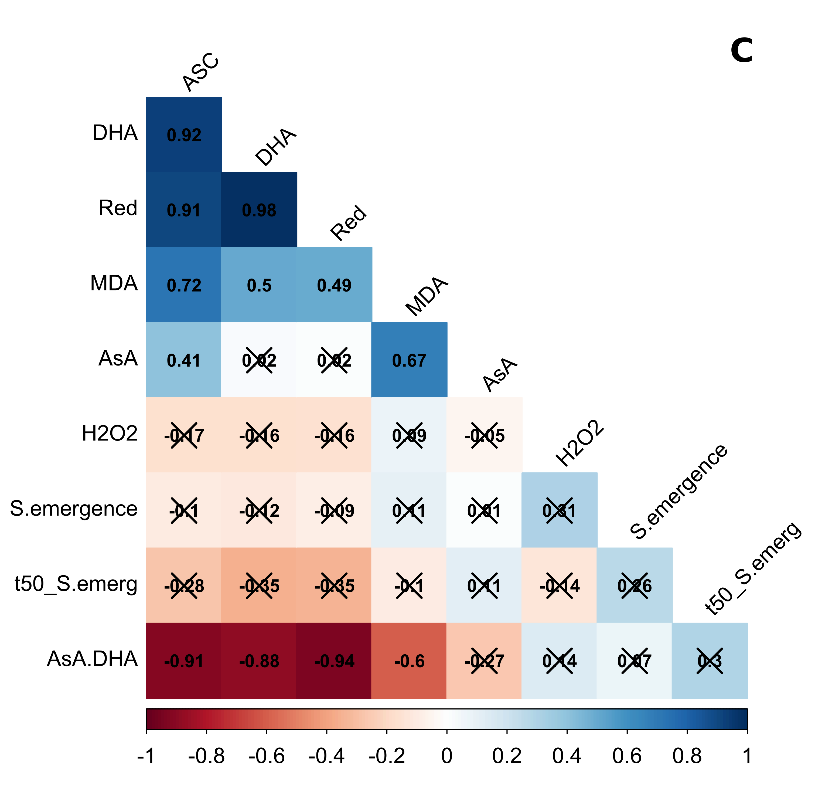


TBARS

TBARS

TBARS

TBARS

TBARS

TBARS

**S4 Fig Correlation matrices for seeds of *P. padus*** stored for two years (A), three years (B), roots (C) and leaves (D) of 3-month old seedlings. Crossed numbers indicate non-significant correlation (P < 0.05). AsA – ascobic acid concentration; AsA.DHA – AsA and DHA ratio; ASC – ascorbate concentration; DHA – dehydroascorbate concentration; Ger. ‑ germination; H2O2 – hydrogen peroxide ; TBARS - thiobarbituric acid reactive substances; Red. – redox potential; S. emergence – seedling emergence; t50_Ger – time to reach 50% of final germination; t50_S.emerg. – time to reach 50% of final seedling emergence.
