# Supplementary material for: Oxidation processes related to seed storage and seedling growth of Malus sylvestris, Prunus avium and Prunus padus
Source: PLoS One. 2020 Jun 18;15(6):e0234510. doi: 10.1371/journal.pone.0234510 (PMC7302524; doi:10.1371/journal.pone.0234510)
Supplement: S5 Fig — (DOCX) [file pone.0234510.s007.docx]

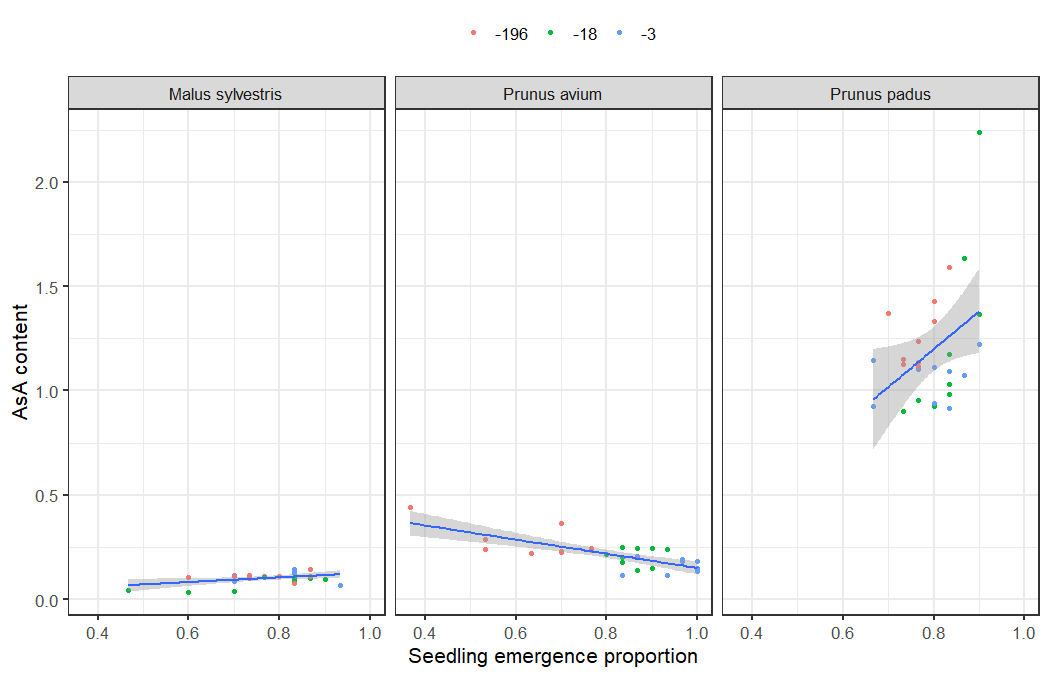


R = 0.44

*p* = 0.022

R = -0.74

*p* < 0.000

R = 0.41

*p* = 0.033

**S5 Fig.** Correlation between seedling emergence and AsA content of *M. sylvestris, P. avium* and *P. padus* seeds after three years storage in different temperature.
